# Supplementary material for: Causal Relationship between Aging and Anorexia Nervosa: A White-Matter-Microstructure-Mediated Mendelian Randomization Analysis
Source: Biomedicines. 2024 Aug 16;12(8):1874. doi: 10.3390/biomedicines12081874 (PMC11351342; doi:10.3390/biomedicines12081874)
Supplement: Supplementary file 1 [file biomedicines-12-01874-s001.zip › Supplementary Figure/Figure S8/Leaveoneout plot of MR analysis for TL to ubm-b-2099.pdf]

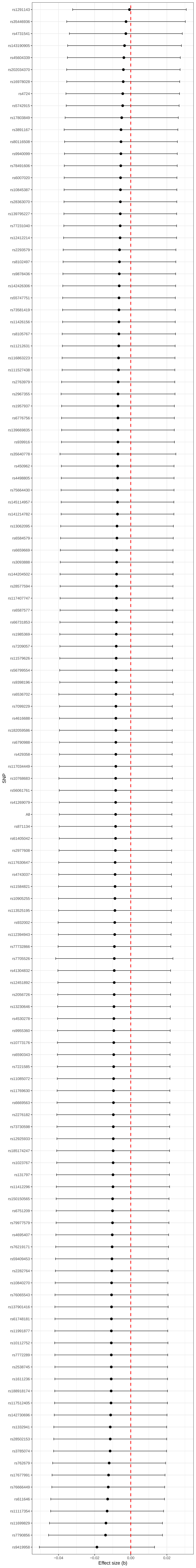
rs1291143
rs35446936
rs4731541
rs143190905
rs45604339
rs202034370
rs16978028
rs4724
rs5742915
rs17803849
rs3891167
rs80116508
rs9940099
rs78491606
rs6007020
rs10845387
rs28363070
rs139795227
rs77231040
rs12412214
rs2293579
rs8102497
rs9878436
rs142426306
rs55747751
rs73581419
rs11426156
rs8105767
rs11212631
rs116863223
rs111527438
rs2763979
rs2967355
rs1957937
rs6776756
rs139669835
rs939916
rs35640778
rs450962
rs4498805
rs75664430
rs145114957
rs141214782
rs13062095
rs6584579
rs6659669
rs3093888
rs144204502
rs28577594
rs117407747
rs6587577
rs66731853
rs1985369
rs7209057
rs11579626
rs56799554
rs9398196
rs6536702
rs7099229
rs4616688
rs182059586
rs6790988
rs429358
rs117034449
rs10768683
rs56061761
rs41269079
All
rs871134
rs61405042
rs2977608
rs117630647
rs4743037
rs11584821
rs10905255
rs113525195
rs932002
rs112394943
rs77732866
rs7705526
rs41304832
rs12451892
rs2056726
rs13230646
rs4530278
rs9955360
rs10773176
rs6590343
rs7221585
rs11085072
rs11769630
rs6669563
rs2276182
rs73730598
rs12925933
rs185174247
rs1023767
rs131797
rs11412296
rs150150565
rs6751209
rs79977579
rs4695407
rs76219171
rs59409453
rs2282764
rs10840270
rs76065543
rs137901416
rs61748181
rs11991877
rs10112752
rs7772289
rs2538745
rs1611236
rs188918174
rs117512405
rs142730696
rs1332941
rs28502153
rs3785074
rs762679
rs17677991
rs76666449
rs611646
rs11117354
rs11699829
rs7790856
rs9419958
SNP
−0.04
−0.02
0.00
0.02
Effect size (b)
